# Building boundary-crossers in clinical-translational research: An exploratory study of a novel communication intervention

**DOI:** 10.1017/cts.2025.78

**Published:** 2025-04-28

**Authors:** Carrie Cameron, Irene Rull-Garcia, Laura G. Meyer, Jose-Armando Fernandez-Guerrero, Erin K. Dahlstrom, Christine Bell, Christine Pribbenow

**Affiliations:** 1 The University of Texas MD Anderson Cancer Center, Houston, TX, USA; 2 University of Innsbruck, Innsbruck, Tirol, Austria; 3 University of Colorado, Denver, CO, USA; 4 Emerson University, Boston, MA, USA; 5 Wisconsin Center for Education Research, UW-Madison, Madison, WI, USA

**Keywords:** Research training, communication skills, mentoring, clinical-translational science, near-peer mentors

## Abstract

**Introduction:**

Integrating scientific research across multiple disciplines to advance breakthroughs is at the heart of clinical-translational science (CTS); among competencies that have been identified as essential for progress, skillful communication is critical. Few tools are available to address the social dynamics of the multidimensional diversity characteristics of CTS. We created the “Building a Diverse Biomedical Workforce Through Communication Across Difference (CAD)” workshop intervention. Based on principles of intercultural communication, CAD taught novel situationally-based communication skills to dyads of near-peer mentors and their undergraduate mentees. This study reports on the effectiveness of the operative mechanisms employed in CAD workshops for helping participants navigate highly diverse research environments.

**Methods:**

Participant data were collected from multiple sources, including workshop artifacts as well as focus groups conducted post-workshop. Data were organized, individually coded, and then iteratively and collectively into pre-defined and emergent themes.

**Results:**

Responses indicated that the content and activities resonated strongly with participants and illuminated their understanding of challenges (both their own and others’) related to belonging, confidence, and connectedness to the research environment; several participants shared that they planned to use or had successfully used the skills. Focus group comments revealed that participants recognized the potential of the skills to include significant opportunities for non-instrumental interaction, contributing to a psychologically healthier workplace.

**Conclusion:**

A brief intervention to develop communication skills across a variety of differences characteristic of clinical-translational settings improves communication between mentors and mentees and with peers and increases sense of belonging in the workplace, with potential benefits to wellbeing.

## Introduction

Integrating scientific research across domains and disciplines to advance scientific breakthroughs is at the heart of clinical-translational research. The complexity of the clinical-translational environment demands new approaches and skillsets to enable diverse, highly-specialized professionals to harmonize disparate goals, methodologies, and disciplinary and professional cultures within multi- and transdisciplinary teams[1]. A consensus statement issued by the Clinical Translational Science Award (CTSA) Research Education and Career Development committees outlines key desired characteristics of a new training and education environment capable of producing a “qualitatively different researcher” [2]. Among seven critical research competencies proposed in the consensus statement as essential to successful work in CTS, three are highly relevant to navigating cultural complexities: boundary-crossing or -spanning (“break[ing] down disciplinary silos and collaborat[ing] with others across research areas and professions”), skillful communication, and teamwork. Of these, skillful communication is the most foundational, since boundary-crossing and teamwork rely on it.

As cultures, these disciplines and domains have social structures, values, traditions, and behavioral norms. Disciplinary cultural differences result in barriers for interdisciplinary work including “problematic plural values” (epistemic and ethical value differences between disciplines)“ [3], pragmatic incoherence” (lack of a path of action due to gridlock of multiple points of view and values) [4], and “disciplinary policing” [3] (also known as “disciplinary chauvinism”). Communication style plays a role as well. Beyond simple differences in the meaning of particular scientific terms, disciplinary patterns of communication emerge [5] which are even more difficult to surface and parse than terminological semantics.

In addition to disciplinary and ethnocultural differences, researchers in training also encounter differences in native language, in institutional backgrounds (size, student body, amount and type of resources, mission focus [university, professional or technical school], level of prestige); in generational status; and in relative power associated with career stage (mentor, mentee; student, postdoctoral fellow, faculty).

Researchers training in the CTS environment experience these multiple dimensions simultaneously but receive little guidance in making sense of them. Environments in which the co-existence of multiple cultures remains unacknowledged and unaddressed are often characterized by ambiguity and uncertainty [6]. (Ambiguity is a characteristic of situations; uncertainty is the affective response to ambiguity [7].) In turn, high levels of ambiguity and uncertainty can result in psychological distress, including avoidance, anxiety, and confusion [8]. As an example, impostor feelings, or uncertainty about belonging, have been associated with psychological distress among STEM and medical students [9,10], and have been found to intensify rather than lessen over time [11]. Chakraverty, Cavazos, and Jeffe reported that MD/PhD students experienced moderate-to-intense levels of impostor feelings, struggling with professional identity formation and fear of evaluation, all of which were particularly acute for those from under-represented groups [12]. Addressing communication across difference may help to mitigate these feelings through reduction of uncertainty.

The “Building a Diverse Biomedical Workforce Through Communication Across Difference” project (CAD, U01 GM132219) studied social and psychological effects of an innovative communication skills workshop on emerging STEM researchers, examining dynamics of interactions among dyads of PhD or postdoctoral near-peer mentors and their undergraduate mentees actively working together. Using a multidimensional, intercultural approach, CAD workshops taught awareness and skills for making sense of the social complexities of the research environment. We use the term ‘intercultural’ broadly, as referring not only to ethnicity but to any large organized group, including disciplines, professions, etc.

The current report, a sub-study of the broader CAD study, focuses on the operative mechanisms used in CAD. Few current approaches for communication skills development are equipped to address multidimensional, situationally-based communication dynamics; new, “qualitatively different” approaches are needed. To study whether and how well CAD’s innovative mechanisms raised participants’ awareness, perceptions, and skills, we collected and analyzed data from both the workshop itself and from subsequent focus groups.

## Conceptual framework

Within a framework of language and social psychology (LSP) [13–16] which centers affect, cognition, and motivation as key drivers of communication dynamics, the CAD study examined variations in *situational communication styles and strategies*, assuming that individuals modify their communicative strategies depending on their relative relationship to conversation partners and the situation itself. These styles are often associated with ethnocultural background but are characteristic of other kinds of groups as well, such as professions and disciplines (e.g., research vs. medicine). Within the LSP framework, the *intercultural communication* approach taken by CAD includes groups that are not ethnic, such as disciplines and organizations, and is pragmatically oriented, concerned with the development of skills to manage interactions successfully (i.e., with mutual understanding) [13]. Communication styles are viewed as patterns, not as personality or ethnic types [17], with much more situational variability and flexibility than types. Rather than answering the question, “What am I like?” or “What is my culture like?,” they answer the question, “How do I (or we) usually try to demonstrate goodwill and respect in this kind of situation?” [17–19] The ability to navigate effectively and appropriately with people from diverse backgrounds across different situations stems from the development of awareness and skills for recognizing and using these styles [18,20] and has been identified as increasingly important for professional and scientific practice in the twenty-first century [21–23].

Three styles commonly involved in interactional ambiguity are directness, formality, and expressiveness. When these style differences are unrecognized or misunderstood in conversation, they can result in uncertainty at best and negative attributions at worst. *Directness* (indirect vs. direct) refers to how openly information is shared, especially information revealing the speaker’s or others’ true attitudes [17,24–26]. Groups using direct style prioritize openness and transparency, even if it results in consequences that may be disagreeable or adverse to the hearer. In an indirect style, tact and discretion are valued as strategies to avoid displeasing the hearer, to show kindness, or to express modesty. The harmony of the relationship is prioritized over explicit, unvarnished detail. The direct style may be perceived as aggressive, boastful, or imprudent to a speaker who uses indirect style, while in the reverse situation, the indirect style may be perceived as passive, passive-aggressive, distant, or even deceptive. While so-called individualist cultures (U.S. culture, for example) are typically characterized as direct compared to others [27], they may exhibit more indirect tendencies in certain situations (for example, impinging on others’ autonomy by telling them “what to do”) [28], causing additional confusion in relationships of unequal power, such as those of mentors and mentees.

*Formality* (formality vs. informality*)* refers to the amount of deference or distance deemed appropriate to display overtly [29,30]. Formality acknowledges hierarchy and/or social distance, while informality conveys familiarity and affinity [27]. A simple example of informality in the research environment could be addressing a supervisor by their first name without explicit invitation to do so, while an example of formality would be addressing them by title and last name, even if the relationship is long-term or the supervisor is a near peer, and even if invited to use the first name. A more subtle example would be whether it is considered appropriate to ask the supervisor directly for an unscheduled meeting. Displays of formality may appear to create social distance, coldness, or an uncomfortable and artificial power imbalance to those who use informal style, while displays of informality to those who use formal style may be interpreted as rash or disrespectful [25,27]. Ambiguity and misunderstanding about formality is common between less and more hierarchical cultures (such as U.S. vs. Asian and the global south [31]; research vs. medicine [32,33]) and those of different social and geographic backgrounds. Because of the inherently hierarchical nature of mentoring relationships, formality differences may surface frequently.

*Expressiveness* (expressive vs. reserved) concerns how much emotion or attitude is considered socially acceptable or wise to convey, regardless of the speaker’s true attitude [34–37]. Examples of expressiveness include displaying effusive praise and compliments, using elaborate or “flowery” language to signal positive attitudes, or displaying emotion (positive or negative) in work situations. A reserved style includes limiting praise, using vague or neutral language to avoid revealing a potentially undesirable attitude, questionable judgment, or excessive self-disclosure. Those using reserved style may perceive expressiveness as overwhelming, inappropriate, or unprofessional, while those using expressive style may perceive reserved style as cold, unresponsive, or disingenuous.

## CAD workshop design and methods

### Workshop design

CAD included units of brief didactic presentation, group discussion, and breakout activities addressing 1) disciplinary, institutional, and training stage differences and 2) ethnocultural/social background differences. For the first unit, examples of disconnects in communication between those of different disciplinary and institutional background were presented. After large-group discussion, mentor-mentee breakout groups were asked to relate to each other their educational and training path to their current position, including explaining how they ended up studying what they were currently studying and why any changes were made along the way; they also talked about what common misconceptions of their discipline or institution they encountered (example: “my friends think epidemiology is the study of skin diseases”). For the ethnocultural unit, three major well-studied patterns were selected for the CAD workshop: indirectness vs. directness, formality vs. informality, and expressiveness vs. reservedness. These were chosen based on the frequency with which they appear in everyday interaction, as well as their likelihood of triggering miscommunication across differences. Facilitators emphasized that these patterns exist on continua and that their use is situation-dependent.

In addition to these communication patterns, the CAD workshop also incorporated, in a less structured fashion, facilitated discussion of the situational dynamics between individuals of different types of scientific discipline studied, academic institutional background (i.e., research-intensiveness, prestige, size, private or public), and rank or stage of training. For disciplinary differences, CAD introduced illustrative quotes and examples of value differences that researchers might commonly experience. As one example, a quote from a *Nature* article described challenges arising in an economics/climate-change collaboration due to economists valuing simplicity in modeling and climate change scientists valuing complexity in modeling, resulting in a lack of trust in each other’s methods. An example at the student level involved challenges in a collaboration between architecture and biology students resulting in disagreements about scheduling and timeline planning of procedures due to their respective work demands. These examples were designed to allow participants to surface their own experiences in encountering disciplinary differences, even if they were not currently involved in a specific project.

When describing common communication patterns in the CAD workshop, no group (in terms of research domain, scientific discipline, institutional culture, stage of training, or ethnicity, etc.), was positioned as a reference group. This design was intended to ensure that dialog about differences felt safe for all without privileging any particular group. Participants were encouraged to recognize communicative differences and interpret them as well-intentioned behavior, not to use them to predict how others might behave or feel.

### Participant recruitment

To ensure a highly diverse environment for learning about intergroup communication dynamics, participants were recruited from a national sample varying in disciplinary focus, ethnicity, nationality, institutional background, and stage of training. Recruiting participants in dyads of undergraduate research mentee and near-peer mentor helped ensure differences in relative power. Recruiting participants who did know each other (the mentor-mentee dyads) and who did not (other workshop co-participants) provided additional variability. Dyads from a variety of institutions were recruited through emails to training program directors and administrators or through announcements on social media and mailing lists such as the National Research Mentoring Network’s regular email communication.

### Facilitator recruitment and training

Each workshop was led by 2 trained facilitators at the doctoral through early-career faculty stage and represented a variety of scientific disciplines; institutional backgrounds; ethnicities; linguistic backgrounds; nationalities; and sexual/gender identities. Facilitators were recruited through the investigators’ networks of institutions, at the Texas Medical Center and New Mexico State University, as well as through referrals from those individuals and from the Howard Hughes Medical Institute Gilliam Fellows’ program. All facilitators were trained by the project team; training included rehearsal. Facilitators were generous in sharing ideas and tips with their colleagues.

### Workshop structure

The CAD workshop was divided into two interactive sessions of about 2 hours in duration, delivered through videoconferencing and occurring 3–4 weeks apart. Because the second session concentrated more on content of communication and is of limited relevance to the current study, it is not discussed here.

Dyads were asked to attend the workshops together, which allowed them to discuss content topics in pairs and in group discussions. On some occasions, one member of the dyad attended a different session due to last-minute scheduling conflicts.

The workshop opened with a discussion focusing on interdisciplinary communication in the first half and on ethnocultural communication style patterns (directness, formality, and expressiveness) in the second half. Example dialogs expressing the patterns were discussed and explored through brief case studies and sample dialogs. This raised participants’ awareness of the styles, helping them to identify usage of the patterns in themselves and their dyad partners and co-participants and to understand them as style differences rather than as personality traits or attitudes. Importantly, the styles and examples were never explicitly associated with any specific ethnicity. Facilitators emphasized that in such a brief session only a few major aspects of style differences could be explored and that the workshop content was intended to complement rather than redefine principles of diversity, equity, and inclusion presented in other types of workshops. Typically, dyad partners had one or more differences in background to discuss, but they also benefitted from hearing the other participants and facilitators talk about their own experiences.

## Study methods

### Data collection

Data for this study were collected from participant artifacts such as Google-doc workbooks, Zoom chat logs, and workshop evaluations during and immediately after the workshops. Additional data were collected approximately four months post-participation from three focus groups and one interview. Copies of the workshop evaluation form and the focus group discussion guide are included in supplementary online material.

Administration of informed consent (MDACC IRB 2019-2010 MOD009) to collect qualitative data from workshop artifacts was initiated in June of 2022. For participants who participated in workshops from June 2021 to February 2022 (91 individuals), retrospective informed consent was collected by emailing the former participants. In cases where 100% of a workshop’s participants did not provide informed consent, all anonymous data were excluded. Participants were not compensated.

In-workshop qualitative data were compiled by the project manager (EKD) from Zoom recordings and data files and from free responses on evaluation forms.

Focus groups were conducted by a trained facilitator from the Wisconsin Center for Education Research via videoconference (UW IRB Exempt under 45 CFR 46, Submission ID: 2022-1679; informed consent: MDACC IRB 2019-2010 MOD009, 12/15/2022). Groups for mentees only and for near-peer mentors only were held from February 6 to 22, 2022, a minimum of thirteen weeks after workshop participation. Focus group participants received an Amazon gift card for $20 as compensation.

The focus group facilitator transcribed the conversations and provided the transcript to the research team for analysis.

All data were segmented and entered into Excel (EKD, IRG), and included the data source (workbook, chat, evaluation, or focus group), date, participant ID number where available, and role (**N**[ear] **P**[eer] **M**[entor], **S**[tudent]) where available. Some comments extracted from workbooks could not be linked to specific participants.

### Data analyses

To investigate the mechanisms driving participants’ response to the workshop content and discussions, a team of five team members including the program director (CC), qualitative methodology specialist (CP), and workshop facilitators (IRG, LGM, JAFG) formed the data analysis team. After familiarizing themselves with the data, they hand-coded it according to pre-defined themes addressed directly by the workshop (ethnocultural differences including directness, expressiveness, and formality; disciplinary differences). In addition to the pre-defined themes, emergent themes were identified. The team engaged in multiple rounds of peer-checking.

Both deductive and inductive methods were used for analyses of the qualitative data. Initial thematic analysis used pre-defined categories that reflected the dimensions of difference explicitly targeted in the content of the workshop: ethnocultural, disciplinary). Emergent themes were identified inductively. These reflected participants’ more general thoughts and feelings about the culture of research that arose from the workshop discussions.

## Results

### Participants

At the conclusion of data collection (February 22, 2023), 126 participants (63 dyads) had attended the workshops. Data of any participant prior to February 2022 who did not provide express retrospective informed consent was excluded from analysis, a total of 16 individuals (10 mentors, 6 mentees). All participants who participated from March 2022 to November 2022 provided prospective informed consent to collect qualitative data from workshop artifacts. The total number of participants whose workshop artifact data was included is 110 (53/63 mentors and 57/63 mentees).

Three focus groups, 2 for mentees (6 total participants) and 1 for mentors (4 participants), plus one individual interview with a mentor were completed. Gender and ethnicity of focus group participants are excluded to maintain anonymity.

### Demographics

Reported gender of all workshop attendees included approximately 73% female, 23% male, and 4% not reported. Reported ethnicity of workshop attendees included approximately 18% Latino/a (any race). Reported race included 63% White, 12% African-American or Black, 17% Asian, 8% more than one race or not reported.

### Effects of workshop mechanisms

Overall, participant responses aligned well with the desired effects of the topics and activities selected for the workshop, at times showing greater impact than expected. The topics provoked energetic discussion, and the participants made larger connections to the general culture of STEM research and of the mentoring relationship, both existing and ideal. Participants expressed relief that their uncertainties about role and belonging were normalized and shared by others.

The pre-defined themes and emergent themes are presented below with illustrative excerpts, grouped by mentee (regular font) and near-peer mentor (italic).

### Ethnocultural differences

When asked about what was most surprising in the workshop, many of the participants noted how ethnocultural differences (direct/indirect, formal/informal, expressive/reserved) play a role in their communication styles and interactions. Directness and formality were referenced most often; expressiveness was mentioned, but to a lesser degree. These discussions validated the experiences of many participants, especially those from international or other backgrounds less common in STEM research; those who were from more “mainstream” backgrounds found it eye-opening to hear about new ways to navigate conversations socially which they had not previously recognized. Participants drew clear connections between workshop learnings and their value in the mentoring and peer relationships.

As a direct-style communicator, seeing the impact of indirect instances and how to better facilitate these conversation helps when I encounter these in my own experiences. *Mentee 100*

*Applying the expressiveness, directness, and formality terms to my own mentor and mentee experiences [was helpful]. This helped me frame why some communications have been strange or incomplete in my career and … that it is OK to have different communication styles from others.* Mentor 107

*So someone has to teach [the mentees], there’s uncomfortable parts […] I think that’s something that I’ve really like brought to light a lot in the CAD workshop and has given me a lot of tools to overcome that. […] I do feel like I’ve gotten confidence to deal with those uncomfortable conversations.* Mentor 1

### Differences within and across scientific disciplines

Conversations about navigating disciplinary differences in communication during training were energetic, and many found it validating and normalizing to realize that feelings of being “inexperienced” or an impostor in such situations were more widespread than they realized.

So how I entered *[interdisciplinary biological science*] is, I like, entered it from [*social science*]. I really enjoyed listening to other people’s perspectives regarding communication in academia. I found that it’s really common to not understand the work of people in other disciplines, regardless of your education level. That made me feel less incompetent, considering that I’m really new to research. *Mentee 101*

*Mainly with…advocating more, I think I’ve gained…the confidence to advocate for myself, and…for others more clearly, especially…within my field. I’m technically a [biological science], so like in the [biological science] department. And there are just some people that treat other people not how they should. And before, I just was like, “Oh, okay.” But now, I’ve like had the confidence be like, “okay, this is not like an okay form of communication,” like, there are things that I can do to address this problem.* Mentor 3

*I’m trained as a biologist, but I work in [STEM education], and I do research in [STEM education]. And I feel like, communicating with others, co-writing grants or other things, people will be like, “what do you even do all day?…. And I’m like, “what?” and they are like, “Do you even publish in journals? or have hypotheses?” And I’m just like, “what?...,” I get mansplained to about my field…* Mentor 2

### Emergent themes

Three themes emerged during analysis, which were coded in vivo: “The Actual Humans That We’re Training:” Significance of Non-Instrumental Communication; “I’m Not as Different as I Thought:” Surfacing the Self Normalizes Difference, and “They’re Human Too:” Power Differences and Vulnerability. Beyond uptake of the workshop content, these themes reveal participant attitudes about the culture of STEM research itself and the psychosocial needs of STEM researchers. Many comments indicated how workshop conversations led to ways of improving the mentoring relationship.

### Theme 1: “the actual humans that we’re training:” significance of non-instrumental communication

Many participants stressed the importance of getting to know the dyad partner better and of having conversations that were not limited to work and task completion. Having personal conversations not only helped to make the work environment more hospitable but also further developed mentor-mentee trust and helped them understand their science/research as well.

I really enjoyed talking to my mentor in the breakout room and learning more about him on a deeper level. Understanding his motivations and true interests really helps me see his point of view better and allows me to seek specific advice in the future. *Mentee 196*

*…Essentially what I would want to say to my PI is ..., if you actually want to make a difference, yes, your research like absolutely. But you want to make a difference with the actual humans that we’re training, and the environment which they’re working in. We cannot continue to have this like hard skills and soft skills language. Like the language I think is harmful… And so, for me, that’s what these trainings... legitimize is that, they’re people, not just workers. […] But if my… humanness is invisible, it’s a lot easier to just do things that are shitty, you know, and like that’s justified. So… that’s what’s coming up for me. When people offer trainings on these things, and actually dedicate time during the work week it’s like you’re saying, this is important.* Mentor 2

### Theme 2: “I’m not as different as I thought:” Surfacing the self normalizes difference for trainees

The second emergent theme was labeled, “I’m not as different as I thought.” Here participants shared inner concerns about their own place and sense of belonging in the research environment, with many pointing out the value of being able to hear the experiences of a much broader group of peers, of varying disciplines, institutions, and lab cultures, and on both sides of the dyad. Comments reflect a palpable sense of relief at how much their thoughts and feelings were similar to others.

[I was surprised] that many traits I thought were “my own” problems were actually very common. *Mentee 101*

[I was] able to see so many other pairs of mentors and mentees […] it was almost reassuring to see that through the workshop that everybody has, you know, completely different experiences with their mentor, and that’s perfectly okay. *Mentee 5*

*I do want to say I really appreciate being able to hear from all the other people here which relates to CAD. […] I just feel sometimes like I’m in my own brain. And then, hearing from other people, I’m like, Oh, yeah, a lot of similarities.* Mentor 2

### Theme 3: “They’re human too:” Power differences and vulnerability

One of the most salient themes to emerge from the data was how strongly power differences and hierarchical roles—including between undergraduates and graduate students—inhibited communication and shared understanding. The reflections offered in this theme revealed that despite the participants’ proximity in age and stage of training, power issues elicited many anxieties for the mentees about competence and intelligence and whether they felt it was safe or prudent to ask questions or admit to not understanding. Although near-peer mentors have been found to be more relatable and approachable than faculty mentors [38,39], the power differential felt by the mentees was considerable. At times mentors, despite their intentions to meet mentees where they were, mentioned either being unaware of when or how certain conversations contributed to the power difference, or underestimating the impact of the differences. Discussions about the workshop activities helped both sides bridge this gap. It is notable that power difference per se was not a workshop topic, but emerged naturally from discussions of communication style differences such as directness and formality, with participants drawing these conclusions themselves.

At first, I was pretty intimidated, … and then, at a certain point, it was not helpful for me if I didn’t speak up. So then I told her if she could break it down for me [*sic*], and then that’s what helped me understand the project better. And she switched her mentoring style essentially after that. *Mentee 2*

[I learned] how to approach PhD students as well as faculty, you know, sometimes it can be a very daunting task to go up to them and ask questions or anything. So it was really cool to see like techniques to, you know, overcome that barrier. They’re humans, too. They’re in the same boat as you. *Mentee 6*

*[My mentee] and I discussed our shared experience of going through a period of being afraid to ask for clarification from a mentor due to anxiety about knowing too little.* Mentor 82

*When they opened it up for discussion…a lot of the mentees were kind of not as vocal as I think they would have been if it was just a group of mentees, and just a group of mentors separately.…I noticed that a lot of the people that spoke up were the mentors. And I thought that was really interesting.* Mentor 5

*So, I think that this workshop kinda helps me think like, oh, how would I feel like if I went into a new place, and I didn’t know? And then people were getting mad at me because I didn’t know? So, yeah, I guess it’s just like made me think differently about how to react to things and how to address things.* Mentor 3

Some participants, however, felt that the workshop didn’t go far enough in discussing power distance in the research environment:

*There could have been] a little more consideration of power structures/ possibilities for abuse from advisors. Assuming best intentions is a nice idea, but like there are a ton of PI’s out there who have made their entire careers out of grinding away advisees…Framing every conflict as a failure of communication might diminish the role played by structural problems, power imbalances, and competing material interests.* Mentor 102

These and similar comments revealed the dissatisfaction participants experienced, especially the more experienced mentors, about how power differences are handled in the research environment.

## Discussion

This study offers insights into the dimensions of socio-cultural communication complexity inherent in research and research mentoring in the clinical-translational environment, including ethnocultural, disciplinary, and role-related dimensions, and how they might be addressed to improve communication and potentially increase sense of belonging. CAD’s novel mechanisms for surfacing, articulating, and placing these dimensions in context produced the intended effects, at times at a pace that exceeded the team’s expectations. Researchers are navigating not just disciplinary differences, or racial/ethnic diversity differences, but many differences simultaneously that are difficult to distinguish, resulting in uncertainty and ambiguity. Results show that explicit discussion of these various dimensions within a diverse audience, as well as acknowledgement that they are happening simultaneously and not in discrete, unconnected interactions, can enable participants to make sense of and normalize them. Participants’ reflections highlighted the impact of non-instrumental communication on the mentoring relationship and on their confidence and sense of belonging, as well as sometimes intense wishes for a more humanized research environment. The CAD workshop suggests possibilities for realizing these benefits both vertically in mentoring relationships and horizontally in peer relationships simultaneously.

### Pre-defined themes

The ethnocultural differences of directness, formality, and expressiveness discussed in CAD afforded participants either new concepts or re-interpretations of existing experiences, helping to move from feelings of individual deficits (of self or others) to a more balanced understanding, without reference, crucially, to specific identities. Participants welcomed the discussion of cross-disciplinary communication and its potential to feel threatening and appreciated the opportunity to find out more about others’ work in a non-judgmental environment. Many expressed new confidence about their abilities and qualifications upon hearing that others felt the same doubts about being able to understand the science of other fields. Enthusiasm for hearing about the variety of scientific disciplines of their peers as well as the workshop facilitators and for getting new perspectives on how others do science was evident. Overall the data reflect that the objective of introducing participants to innovative strategies for understanding and communicating across differences was achieved, with positive results.

### Emergent themes

The emergent themes of “I’m not as different as I thought,” and power distance and vulnerability candidly illustrated participants’ inner opinions about life in the research environment. These scholars and future faculty almost uniformly desire more meaningful and non-instrumental social contact with supervisors, supervisees, and peers within and outside of the research team, and they recognize the importance of that contact in their own development. The realization that many are “not as different as they thought” and the descriptions of how power differences affect interaction indicate their potent and mostly unspoken influence on mentoring relationships and sense of belonging in research. The theme of “They’re Human Too” clearly articulated desire for a more hospitable social culture in STEM research and called for mentors to play an active role in building it.

Despite their brevity, the CAD workshops provided participants with vocabulary to articulate observations and questions that are rarely expressed in the research environment, better understanding of and perspective on their own and their peers’ communicative styles, and a framework for navigating a range of differences that applied to their daily lives. Translational science trainings have addressed these skills but have tended to focus on discrete, prescriptive organizational goals such as managing conflict, training leaders, or building teams [29,30] and/or on content-focused research communication skills such as techniques for writing scientific articles, presenting research, and reducing jargon [31]. Issues related to interdisciplinary and intercultural communication, however, benefit from being considered together as part of the same discovery process: recognize that out-of-awareness differences in point of view exist, surface and explore them, understand their impact on interaction [40,41]. Like ethnic cultures, disciplinary cultures transmit values, norms, and behaviors through training and socialization [43], and evidence suggests that positive attitudes to intergroup collaboration, including multidisciplinary work, can be fostered by instructors who model positive attitudes to such collaboration [44]. Socialization is at the heart of the mentoring relationship and thus fundamental to training boundary crossers in translational science.

The intercultural, multidimensional approach taken by the CAD project allows participants to directly address research culture and practices that relate to them and their concerns and to understand that their impostor feelings and doubts about belongingness are shared by others, thus normalizing them. At the same time, they are better able to see the humanity of their peers. These results show that interventions like CAD, with its novel focus on the nature and social-psychological dynamics of communication, may be useful for addressing impostor feelings, belongingness, and associated psychological distress.

Limitations of this study include a relatively small sample size and potential self-selection bias in the focus groups. The 13-week interval from participation to focus group invitation, combined with persistent disruptions to educational programs due to COVID-19 as well as normal separation of dyads upon project completion or graduation may account for the relatively low number of acceptances of the focus-group invitation. Participant comments taken from the workshop artifacts were elicited in some cases by specific prompts (“What was the most helpful part of the workshop?”).

The findings of this study have important implications. First, CAD’s novel situational and multidimensional approach suggests that its design and operative mechanisms can offer an efficient means for increasing sense of belonging in research training and mentoring. Second, as others have shown, positive and supportive social environments in research training are associated with mental health, well-being, and stress reduction [32], currently of urgent concern in STEM research training; CAD creates an environment that helps reduce uncertainty and impostor feelings in communication. Third, CAD directly responds to the complex communication demands of the translational research environment. Because it involves a minimal investment of time and resources and its impact can be seen almost immediately, CAD is highly accessible and cost-effective and is appropriate for use in small groups, such as labs to larger groups, such as departments, organized professional development series, and other settings. While the dyadic format is ideal, non-dyadic versions can be effective as well.

Future study will examine the longer-term effects of CAD participation on social-cognitive outcomes and intention to remain in research careers and to mentor across difference.

CAD represents a new and promising approach for strengthening identity development, mentoring relationships, and skillful communication in complex, highly diverse environments. In the words of one focus group participant, “As someone that works with mentees of all different genders and backgrounds, [CAD] is a path forward that could enhance the work environment…. it’s not just like a sport I’m playing, communication is really integral to the success of our future workforce.”

## Supporting information

Cameron et al. supplementary material Cameron et al. supplementary material

## Supplementary material

The supplementary material for this article can be found at https://doi.org/10.1017/cts.2025.78.
