# Supplementary material for: Building boundary-crossers in clinical-translational research: An exploratory study of a novel communication intervention
Source: J Clin Transl Sci. 2025 Apr 28;9(1):e107. doi: 10.1017/cts.2025.78 (PMC12171905; doi:10.1017/cts.2025.78)
Supplement: Cameron et al. supplementary material [file S2059866125000780sup001.pdf]

Communicating Across Difference Workshop #1 Evaluation

Thank you for participating in the first of two Communicating Across Difference workshops, and for your willingness to complete this short evaluation.We estimate this evaluation will take ~7 minutes to complete. Please note that this evaluation is anonymous, and all information you provide will be kept confidential. Evaluation data will be used to improve future iterations of the workshop.If you have any questions, please contact the Communicating Across Difference (CAD) Team at CAD@mdanderson.org.

Section 1. Demographics (Optional)

How would you describe your ethnicity?

____

What is your title/role?

◯ Undergraduate student
◯ Graduate student
◯ Postdoctoral fellow
◯ Other

If you selected "other," please specify your title/role.

____

What discipline/domain do you study or work in now?

◯ Basic Science
◯ Applied Science
◯ Biomedical/ Bioengineering
◯ Population Science
◯ Clinical Science
◯ Social Science
◯ Other

If you selected "other," please specify what discipline/domain you study or work in now.

____

Section 2. Workshop Evaluation

Which Workshop #1 did you attend?

◯ Tuesday, February 8; 10:00AM - 12:00PM Central
◯ Wednesday, February 9; 3:00PM - 5:00PM Central
◯ Thursday, February 10; 11:00AM - 1:00PM Central

Was the workshop interesting to you?

◯ 1, Not at all ◯ 2 ◯ 3 ◯ 4 ◯ 5 ◯ 6 ◯ 7 ◯ 8 ◯ 9 ◯ 10, A lot

Was the online format of the workshop effective?

◯ 1, Not at all effective ◯ 2 ◯ 3 ◯ 4 ◯ 5 ◯ 6 ◯ 7 ◯ 8 ◯ 9 ◯ 10, Very effective

Was the methodology and structure of the workshop effective?

◯ 1, Not at all effective ◯ 2 ◯ 3 ◯ 4 ◯ 5 ◯ 6 ◯ 7 ◯ 8 ◯ 9 ◯ 10, Very effective

Was the balance between facilitator presentation and audience participation effective?

◯ 1, Not at all effective ◯ 2 ◯ 3 ◯ 4 ◯ 5 ◯ 6 ◯ 7 ◯ 8 ◯ 9 ◯ 10, Very effective

Was the benefit from the workshop worth the time it took?

◯ 1, Not at all worth it ◯ 2 ◯ 3 ◯ 4 ◯ 5 ◯ 6 ◯ 7 ◯ 8 ◯ 9 ◯ 10, Very worth it

Would you encourage your friends and colleagues to participate in this workshop?

◯ 1, No, not at all ◯ 2 ◯ 3 ◯ 4 ◯ 5 ◯ 6 ◯ 7 ◯ 8 ◯ 9 ◯ 10, Yes, absolutely

Should the workshop be shorter, the same length, or longer?

◯ 1, Much shorter ◯ 2 ◯ 3 ◯ 4 ◯ 5 ◯ 6 ◯ 7 ◯ 8 ◯ 9 ◯ 10, Much longer

Please use this space to explain or comment on any of your responses to the questions above.

Which elements of the workshop were MOST helpful to you?

Which elements of the workshop were LEAST helpful to you?

What's the most surprising or eye-opening thing you learned today?

Any final observations, suggestions, or feedback about the workshop?

Workshop Attendee Import

Attendee email

____

Workshop #1 date

____

Workshop #2 date

____

ID #

____

CAD FG Discussion Guide
2/2/2023

CAD is: an intervention that is designed to allow students and near-peer mentors to 'see' other researchers as multi-faceted, whole people and scientists, and to provide skills that are flexible and portable across many situations.

CAD goals: To study the effects of an intervention in communicating across difference on research students (undergraduate, postbaccalaureate, or Master's level) and their near-peer mentors (defined as PhD level graduate students, postdoctoral fellows, or instructors) and their intention to remain in research careers. For near-peer mentors, to study effects on their intention to mentor diverse trainees in the future.

<u>RESEARCH QUESTIONS</u>:

What takeaways do participants have from workshops overall?

- Cultural
- Disciplinary
- Institutional/stage of training
- Communicating with family and friends

What values and wishes about life in the Research Environment [RE] are expressed?

Introduction

Welcome to the CAD focus group, you are all mentors/mentees that participated in the CAD program. Thank you all for participating in the workshops, the survey research study, and signing the consent form with MD Anderson to participate in this recorded discussion today.

Since this is a group discussion, we cannot guarantee confidentiality, but we can all agree to not share the names and identities of others in this virtual room today, or "take ideas with you but leave names and identities here". As a reminder, I am audio recording and saving the zoom auto-transcript from this discussion for accuracy, but the only data that will be shared with the CAD team is a de-identified transcript.

You do not have to answer any questions you do not feel comfortable with. I am going to ask a few questions to the group and feel free to speak up when you have a response, respectfully comment on responses from others if you have something to add or use the chat or hand raise function if you do not want to interrupt what someone else is saying. Due to time constraints, you may not all get to answer every question.

Do you have any questions before I begin recording?

1. We will start with discussing what you remember about the workshop, what were some of your takeaways from the CAD workshops overall?

2. What have you learned, or what have you realized about the **research environment** since participating?
    a. What aspects of the research environment do you pay more attention to now, after the workshops?

3. What have you learned, or what have you realized about your **mentor and/or your peers** in research training since participating?
    a. Have you been more aware of their communication styles? In what ways?

4. What have you learned or what have you realized about **yourself** since participating?
    a. In what ways do you see yourself as a multi-faceted, whole person scientist?

5. Some of the themes of the workshop are: **culture, discipline, institution, stage of training, and communicating with friends and family**. Are any of these especially prominent for you personally?
    a. Were there any other themes from the workshop that were relevant to you?
    b. How has the theme of communicating with friends and family been relevant to you?

6. What reflections did workshop participation prompt for you about life in the research environment?

7. What, if anything, **has been different for you** since the workshop (even if it's just a perception)?
    a. Has your satisfaction or comfort in the research environment changed? In what ways? Why?

8. Would you **change anything about the research environment** based on your experience with the workshops? (even if it's just a wish and not 'realistic')

9. Would you **change anything about the workshop** content, looking back? [optional, if time]

10. As a result of participating, what would you like the PI [NOT near-peer mentor] of your research experience to know, looking back?
    a. For example, what should your PI know about what life in the research environment is like for you and your lab mates? What is most enriching or positive about lab life?

11. As a result of participating, what would you like others who are **beginning in research** or **beginning to mentor** to know, looking back?
    a. Based on your experience, what could people be more open about? What would you change about the way you get along or have relationships in the lab?
    b. If you could give some advice about these themes to the next person, what would you say to make them feel more at home when they join the lab?
